# Taking Inventory of a Nervous Case; and the Effects of Season upon the Production of Nervous Diseases1Read at the thirty-second annual meeting of the Medical Association of Central New York, at Syracuse, October 17, 1899.

**Published:** 1900-07

**Authors:** William C. Krauss

**Affiliations:** Buffalo, N. Y., Consulting neurologist, Buffalo General Hospital; attending neurologist, Erie County Hospital, German Hospital, German Deaconess’s Home, and Woman’s Hospital


### TAKING INVENTORY OF A NERVOUS CASE; AND THE EFFECTS OF SEASON UPON THE PRODUCTION OF NERVOUS DISEASES.[1]

By WILLIAM C. KRAUSS, M. D., Buffalo, N. Y.,
Consulting neurologist, Buffalo General Hospital; attending neurologist, Erie County Hospital, German Hospital, German Deaconess's Home, and Woman's Hospital.

DISEASES of the nervous system are indicated by symptoms, objective as well as subjective, which require at times more than the ordinary skill and ability on the part of the physician to be correctly diagnosticated. The minor symptoms are generally so obscure and complex that some plan or procedure is necessary to detect their presence and to correctly interpret their value and significance. A system which will classify the minor phenomena of the subjective and objective major symptoms, will also go far toward unmasking many nervous diseases and render their diagnosis easier and more clearly to the student and practitioner.

The purpose of this paper is to present just such a system, and if followed out cannot fail to accomplish the purpose for which it was intended.

A successful business man has a system in taking inventory of his stock and when completed he is in no doubt as to his financial condition. A physician who employs a like method in inventorying a nervous patient arrives at the same satisfactory result and can proceed in the treatment of his patient in a thoroughly rational and scientific manner.

*Anamnesis:*

1. Date or season.
2. Name.
3. Sex.
4. Age.
5. Nativity or nationality.
6. Occupation.
7. Family history { Direct. / Collateral.
8. Early History { Environment. / Development. / Habits. / Characteristics. / Infantile Diseases, including injuries.
9. Present History { Cause. / Onset. / Progress. / Complication.

*Status Presens:*

1. Height.

2. Weight.

3. Expansion.

4. Constitution and habitus.

5. Complexion.

6. Stature.

7. Gait.

8. Posture.

9. Head { Skull. / Scalp.

---

1. Read at the thirty-second annual meeting of the Medical Association of Central New York, at Syracuse, October 17, 1899.

10. Face

    - Symmetry or asymmetry.

    - Tics and Squints.

    - Facial angle.

    - Degenerative stigmata.

    - Expression or facies.

    - Skin and appendages.

    - Color.

    - Vaso-motor disturbances.

    - Painful points.

    - Special Sense organs.

        - Eyes.

        - Ears.

        - Nose.

        - Mouth.

    - Speech.

11. Neck

    - Size.

    - Motility.

12. Body and Extremities

    - Tremor.

    - Spasm.

    - Muscular atrophy.

    - Tendon reflexes.

    - Paralyses.

    - Sensation.

13. Pulse.

14. Temperature.

15. Blood.

16. Urine.

17. Stomach contents.

18. Feces.

19. Mentality.

20. Coma.

### SEASON.

The effect of season upon the production of nervous disorders is well recognised and has been the subject of much inquiry and investigation. Not only do the changing seasons encourage and nurture nerve disturbances, but the changing barometric pressure exerts its influence also. "Living barometers," or those individuals who can feel the storm several days a coming, are met with in every community and their sensitive nervous organisations rise and fall synchronously with the mercury in the tube of the household barometer.

Mitchell and Catlin have studied the influence of weather upon neuralgic disturbances, and less than half of the cases studied felt unusual sensations upon the coming of an east wind and during its continuance; and of these, two-thirds insisted on their power to predict such change in the weather, while the rest thought that any great change was likely to cause them great pain. An annual neuralgic curve was constructed on the monthly ordinates of neuralgic duration, and a large number of neuralgic attacks were seen to be definitely related to storms, and Mitchell believed that some combination of weather started the pain. At the center of the vast rain area of every storm is a moving space of the greatest barometric depression, known as the storm center, which the rain usually precedes by five hundred to six hundred miles, and before and around the rain is a forerunning neuralgic belt (Mills).

In those seasons where both the thermometer and barometer are low and subject to frequent and rapid changes, as during the winter and spring months, there will be found those affections of an acute inflammatory nature, commonly designated as rheumatic or à frigore. These may affect the sensory nerves, resulting in neuritis the trophic nerves, as in Raynaud's disease, the motor nerve, as in Bell's palsy, and the brain and spinal cord, as in meningitis and acute myelitis. Many of the organic, chronic diseases make their initial appearance during these seasons of varying temperature as, muscular atrophy, locomotor ataxia and paralysis agitans. Certain functional disturbances, especially chorea, hysteria and neurasthenia, are found to occur more frequently in the late spring and early summer months, probably due to seasonable changes and a resulting lowered tone of the central nervous system. The system seems to be at low ebb during these months and exhaustive nervous conditions result.

During the summer and fall months the infectious processes seem to be more prevalent, and are represented by such affections as tetanus, acute anterior poliomyelitis and beriberi. The exhaustive neuroses, especially neurasthenia, are also common, the intense heat and humidity of the summer months being enervative to a marked degree.

Some of the nervous diseases more or less susceptible to seasonal influences are arranged alphabetically, giving the months of greatest occurrence. A classification of these diseases, according to the season, then follow: angioneurotic edema, winter months; apoplexy, cerebral, winter months; Bell's palsy, November, December, January, February and June and July; beriberi, summer months; chorea, March, April, May, June; erythomelalgia, summer months; epilepsy, all seasons; laryngismus stridulus, winter and spring months; locomotor ataxia, winter months; meningitis, epidemic cerebro-spinal, winter and spring months, seldom autumn, never summer; meningitis, tuberculous, winter and spring months; migraine, winter months; muscular atrophy (Duchenne-Aran), winter and spring months; myelitis, acute, winter months; myxedema, winter months; neuralgias, late autumn and winter months; neurasthenia, spring and summer months; neuritis, spring and late autumn; paralysis agitans, spring and winter months; poliomyelitis, acute anterior, summer months; Raynaud's disease, winter months; tetanus, August, especially July to October; tetany, January to May.

Spring
- Bell's Palsy.
- Chorea.
- Hysteria.
- Locomotor Ataxia.
- Meningitis, epidemic cerebro-spinal.
- Meningitis, tuberculous.
- Muscular atrophy (Duchenne-Aran).
- Neuralgia, infacial.
- Neurasthenia.
- Paralysis agitans.
- Tetany.

Summer and Autumn

- Bell's Palsy.

- Beriberi.

- Erythomelalgia.

- Neurasthenia.

- Poliomyelitis, acute anterior.

- Tetanus.

Winter
- Angioneurotic edema.
- Apoplexy, cerebral.
- Laryngismus stridulus.
- Locomotor ataxia.
- Meningitis, epidemic cerebro-spinal.
- Meningitis, tuberculous.
- Migraine.
- Myelitis, acute.
- Myxedema.
- Neuralgias.
  - Intercostal.
  - Sciatic.
  - Infacial.
- Neuritis.
- Raynaud's disease.

371 DELAWARE AVENUE.

